# Minimizing the wiring in distributed strain sensing using a capacitive sensor sheet with variable-resistance electrodes

**DOI:** 10.1038/s41598-022-18265-x

**Published:** 2022-08-17

**Authors:** Hussein Nesser, Gilles Lubineau

**Affiliations:** Mechanics of Composites for Energy and Mobility Lab, Mechanical Engineering Program, Physical Science and Engineering Division, King Abdullah University of Science and Technology (KAUST), Thuwal, 23955-6900 Kingdom of Saudi Arabia

**Keywords:** Sensors and biosensors, Mechanical engineering, Sensors, Soft materials

## Abstract

Strain mapping over a large area usually requires an array of sensors, necessitating extensive and complex wiring. Our solution is based on creating multiple sensing regions within the area of a single capacitive sensor body by considering the sensor as an analogical transmission line, reducing the connections to only two wires and simplifying the electronic interface. We demonstrate the technology by using piezoresistive electrodes in a parallel plate capacitor that create varying proportions of electromagnetic wave dissipation through the sensor length according to the interrogation frequency. We demonstrate, by a sensor divided into four virtual zones, that our cracked capacitive sensor can simultaneously record strain in each separated zone by measuring the sensor capacitance at a high frequency. Moreover, we confirm that by changing the frequency from high to low, our sensor is able to measure the local strain amplitudes. This sensor is unique in its ability to monitor strain continuously over a large area with promoted spatial resolution. This sensing technology with a reduced number of wires and a simple electronic interface will increase the reliability of sensing while reducing its cost and complexity.

## Introduction

Strain sensors are widely applied in smart wearable applications, structural health monitoring (SHM)^1–3^, human motion detection^4,5^, soft robotics^6^, medical treatments^7,8^, and human–machine interfaces^9^. The ability to measure strains at different locations, known as distributed strain sensing, has become increasingly important in modern sensing devices. For example, accurate measurement of human movement is essential for interacting with a virtual environment^10^. For localizing damage in structures, a high spatial resolution is required^11^. Finally, spatial/distributed strain detection is necessary for applications involving shape change (morphing) and/or large surface areas^12^. Spatial coverage (ability to cover a large area) and spatial resolution (ability to detect strain at an accurate location) become very important in such applications^13^.

To date, large-area sensing with high spatial resolution has not been achieved due to serious technical limitations. Table 1 summarizes and compares the properties of most common techniques for mapping strain/pressure across a large area. While most techniques provide spatially discrete data obtained using complex wiring, our technique results in spatially continuous measurement using very few connections. For example, the fiber Bragg grating (FBG), for example, is a popular technique in spatially-discrete distributed strain sensing^14^. Although FBG multiplexing is now a well-controlled technique, FBGs are thermally sensitive. Despite the development of compensation techniques, the wavelength shifts caused by temperature and strain cannot be easily distinguished over large areas. Further, FGBs rely on expensive and bulky interrogation systems. Another solution is electrical strain sensors, which implement a network of independent sensors on a large surface^3,15,16^; however, the number of electronic interfaces is directly related to the number of sensors. The simplest type of electrical strain sensor is the well know piezo-resistive strain gauge. It is know to be highly reliable, but results in complex connections when used in large arrays for monitoring strains over large structures. We presented previously a piezo resistive electrical strain sensor based on double-twisted conductive smart threads comprising a homogeneously and a gradient-coated thread^17^. This system partially solves the sampling issue, but the interpretation of the measurement is difficult. A capacitive tactile-sensor array can help reduce the number of cables and electronic interfaces on a device. Two-dimensional (2D) sensor arrays on a single sheet were created by patterning the electrodes on the bottom and top of the dielectric material into orthogonal columns and rows, resulting in pixels at the intersection^18–21^. Nevertheless, the resolution is limited, and detecting the capacitance variation at a specific pixel requires a complex electronic interface using analog multiplexers^22^, decoders^23^, and capacitance-to-digital converters^24^.

**Table 1:** Comparison between the main available distributed strain-sensing methods.

	Spatially continuous vs. discrete sensing	Physics	Wiring and complexity of interrogation system
FBG	Discrete	Optic	Complex network and expensive optical interrogation systems
**Strain gauge**			
Single	Discrete	Mainly using piezoresistivity	2 wire, simple electrical interrogation
Array		Mainly using capacitance variations when used in arrays	2 × n wires, complex connections
DIC (digital image correlation)	Discrete (at correlation points)	Optical flow	Sensitive to optical conditions and environment Available mainly for surface observation
Transmission line	Continuous	Electromagnetic dissipation	Simple connection (2 wires) with low-cost frequency interrogation systems

If the sensor is coerced to act as an analogical transmission line, multiple sensing regions can be created within the area of a single capacitive sensor body, reducing the connections to only two wires and simplifying the electronic interface^25,26^. Capacitive sensing based on the transmission-line model, as demonstrated by Xu et al., can realize 2D touch detection using a stretchable keyboard^27,28^. Applying the same method, Sonar et al. detected the pressure on pixels using a single-sensor body and a single electronic interface^26^.

Little research research focuses on detecting pressure using the signal attenuation method by integrating resistive electrodes on parallel-plate capacitors without addressing the local strain measurement. However, our study distinguished itself by using the transmission-line method to develop a strain sensor that detects strains in multiple elements along the sensor body. This paper describes our experience with a new distributed sensing system based on a transmission line model where we replace a network of local sensors with one sensor sheet. We demonstrate a distributed strain-measurement technique using a single-sheet capacitive sensor, which minimizes the quantity and complexity of wiring numbers. We are able with our technology to detect strain in multiple points of a large structure with one sensor that operates only with two-wire.

We describe in this paper the mechanism of the transmission line-based sensor and how to benefit from the signal dissipation in a capacitive sensor to localize the strain. We also present the evaluation results and discuss the possibility of using such a sensor to follow the strain location and magnitude. The result shows that the our sensor’s ability to detect all strain features provides an unprecedented advantage in terms of accurate and reliable measurement. Finaly, we demonstrate the sensor advantage by using our one-sheet sensor to detect finger bending and crack locations on an engineering structure.

## Results and discussion

### Signal dissipation and virtual capacitance

Our method relies on measuring the transmission-line capacitance (so-called later “virtual capacitance”), which is affected by signal penetration along the sensor length. It is well known that signal penetration in a transmission-line architecture is highly dependent on electrode resistance. A signal can travel the entire length of a low-resistance electrode even at a high frequency. On the contrary, signal dissipation occurs in high-resistance electrodes, reducing penetration length, particularly at high frequencies. Consequently, creating highly piezoresistive electrodes is the first step to use the transmission-line effect for strain measurement.

**Figure 1:**
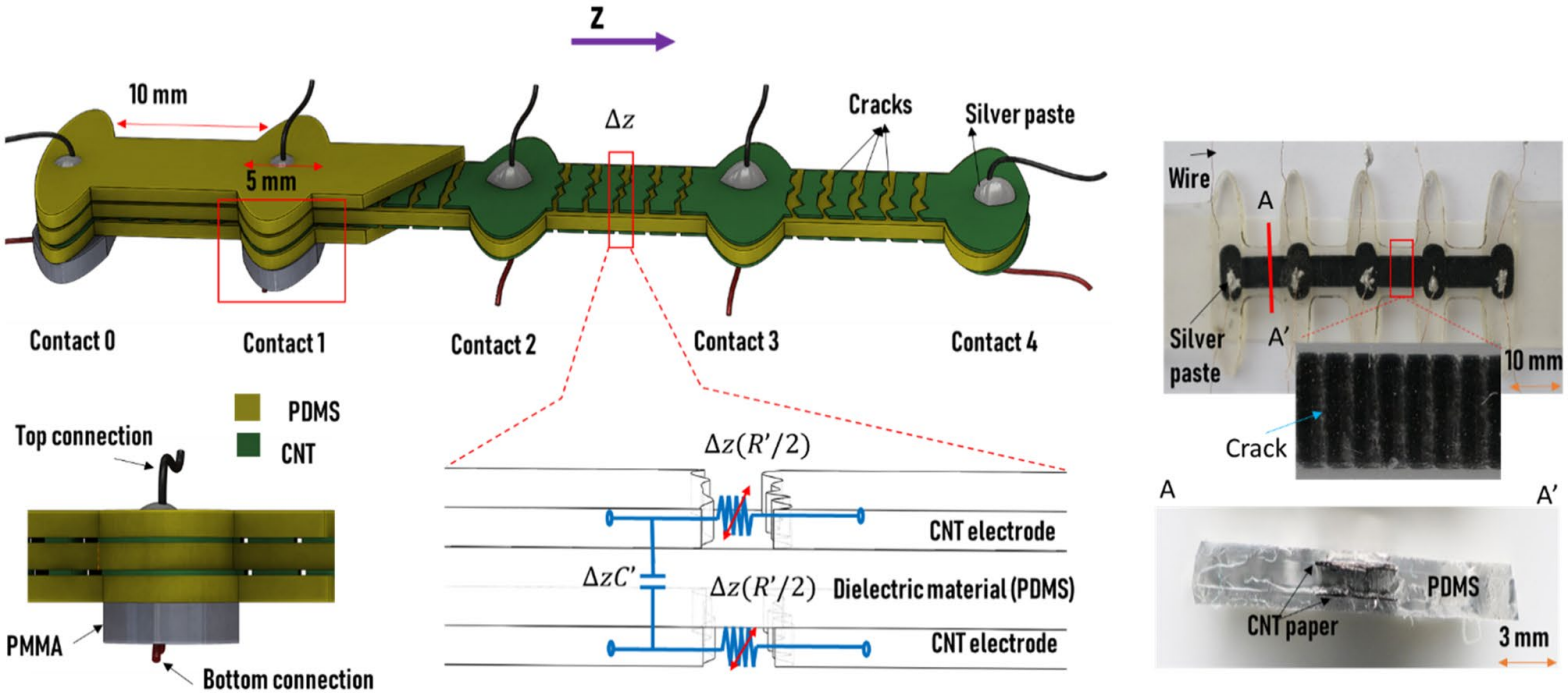
(Top left) Schematic representing the multilayer sensor (the electrical connections are distributed on the top and bottom electrodes at 10-mm intervals); (bottom left) cross-sectional view of an electrical contact section. The bottom of the flexible layers is affixed to a rigid support (PMMA) to ensure nonstretchable electrical connection zones (ellipsoidal breakers); (bottom center) electrical model of a small spatial segment (∆z), where R′ and C′ are the electrodes resistance and capacitance per unit length, respectively; (top right) photographs of the realized structure (with a zoomed-in image showing the cracks); (bottom right) cross-section photograph showing the different layers of the structure.

We consider a one-dimensional stretchable parallel-plate capacitor (Fig. 1) for which the electrodes comprise a single-walled carbon nanotube (SWCNT) film^29^. While SWCNT films are naturally piezoresistive, we amplify the piezoresistivity (resistance–strain relationship) by creating a pattern of cracks^30^. In this configuration, the entangled SWCNT networks within the cracks connect the different nanotube fragments, facilitating the transport of electrons through the electrodes. Under stretching, the SWCNT’s distribution within the crack changes, thereby increasing the electrode’s resistance. The high resistivity of the electrodes that appear after crack opening induces the transmission-line behavior in the entire sensor, mostly at high frequencies. The electrical model of the transmission-line sensor is represented by a chain of resistance–capacitance circuits, where each small segment (∆*z*) is represented by a capacitance ∆*zCʹ* and a variable resistance ∆*zRʹ* (Fig. 1). Herein, $C^{\prime} = C/L = C_{0} /L_{0}$ is the capacitance per unit length and $R^{\prime} = R/L$ is the electrode resistance per unit length*.* The total electrode resistance, *R*, varies with cracks opening along the structure length (Fig. S1). *C* is the total capacitance of the whole structure under stretch and *L* is the length under stretch. Additionally, *C*_0_ is the initial capacitance and *L*_0_ is the initial length.

According to the transmission-line model, the voltage dissipation in the sensor creates a “virtual” sensor length (represented by the distance from the origin to the point where the signal is fully attenuated). Analytically, the virtual length/capacitance is derived from the telegraph equation that defines the voltage attenuation along the transmission line (sensor length) as follows^31^:

1 $$ V(z) = V_{0} e^{{ - \sqrt {\pi fR^{\prime}C^{\prime}z} }} , $$

where *V*_0_ is the magnitude of the alternative input voltage and the exponent is the attenuation factor *α* = $\sqrt {{\uppi }fR^{\prime}C^{\prime}}$. In this expression, *f* is the frequency of the interrogation signal. The effective/virtual length (*L*_eff_) derived from Eq. (1) can be represented as follows^28^:

2 $$ L_{eff} = L \times g(f,R(\varepsilon )) = L_{0} (1 + \varepsilon )g(f,R(\varepsilon )) $$

Equation (2) shows that the sensor’s effective length is affected by a strain-frequency dependent term, *g*(*f*,ε), representing the transmission-line effect.* ε* is the external strain*,* the term (1 + *ε*) represents the geometrical variation under stretching, and *R*(*ε*) is the strain-related electrode resistance (Fig. S1a).

By taking advantage of the linear relation between the structure length and capacitance, the effective capacitance, *C*_eff_, can be obtained as

3 $$ C_{eff} = C_{0} (1 + \varepsilon )g(f,R(\varepsilon )) $$

*g*(*f,R*) ranges from 0 to 1, depending on the variable factors: frequency *f* and strain *ε*. It was concluded in our previous work^29^ as:

4 $$ g(f,R) = \frac{{ - \ln \left( {\frac{{V_{\min } }}{{V_{0} }}} \right)}}{{\sqrt {\pi fCR} }} $$

where *V*_min_ is the voltage at which the transmission line becomes ineffective (the signal does not reach the end of the sensor anymore/the “nonexistent” voltage).

Equation (3) reduces to *C*_eff_ = *C*_0_(1 + *ε*) (the well-known geometrical capacitance variation under strain) when *g*(*f*,*R*) is close to 1 (low frequency *f* and/or low strain *ε*). However, the transmission-line phenomenon dominates at sufficiently high *f* and *ε*, (*g* < 1.0). In this regime, the signal dissipation within the sensor is increasing, decreasing the effective/virtual capacitance.

**Figure 2:**
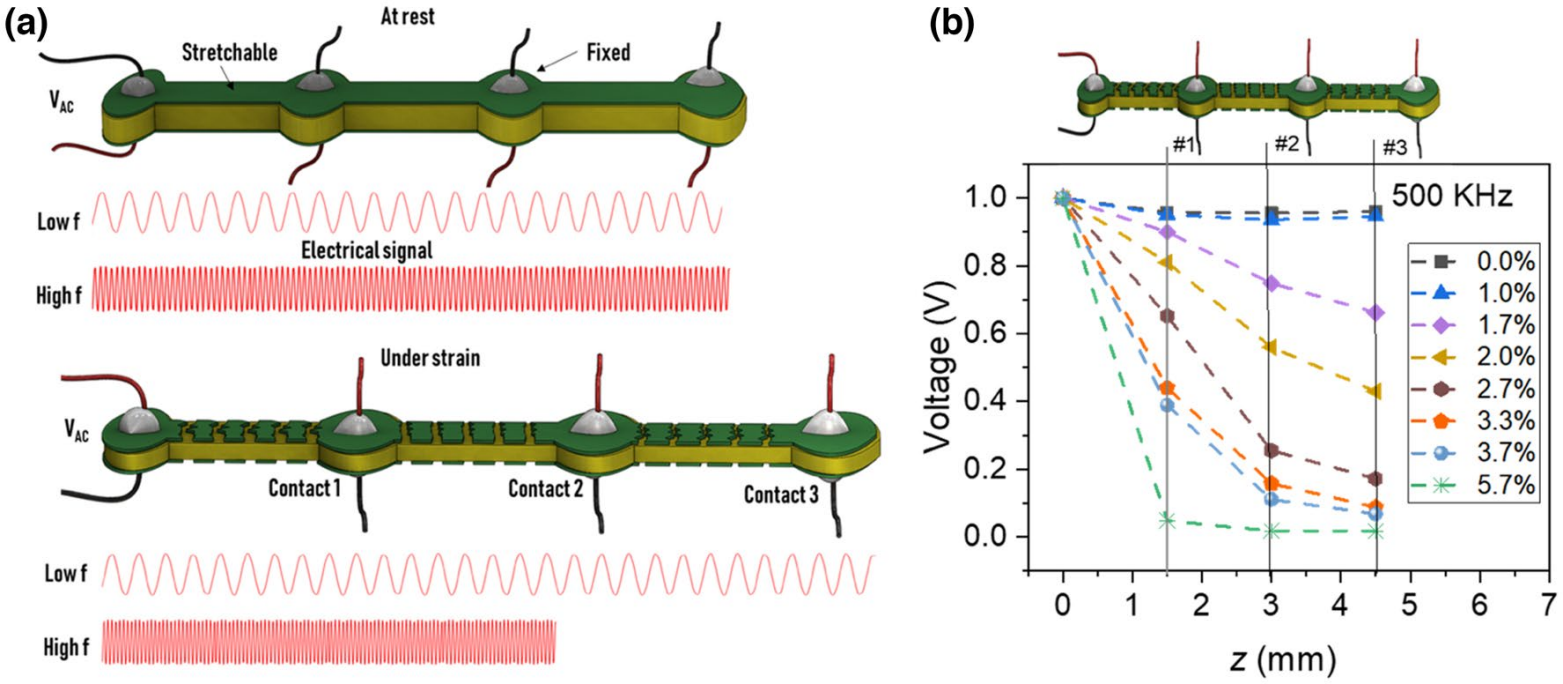
(**a**) Schematic of voltage dissipation through the non-cracked and cracked structure at low and high signal frequencies; (**b**) experimentally determined voltage dissipations along the sensor length (z-direction) under different external strains.

First, we investigated the dissipation behavior of the electrical signals along the sensor length by injecting an alternative voltage at the beginning of the sensor while the voltage was measured at different locations. Figure 2 a,b shows that the signal magnitude was retained along the sensor length under low strains. This signal behavior changed when the strain reached a value of 1% (electrode resistance became noticeable); the signal attenuated before the end of the sensor length. The signal disappeared from contacts #3 (*z* = *L*), #2 (*z* = 2*L*/3), and #1 (*z* = *L*/3) when the strain reached 3.3%, 3.7%, and 5.7%, respectively. The voltage profile was directly related to the strain and decreased progressively as the strain increased. The detailed result of the voltage dissipation through the structure is presented in Fig. S2.

**Figure 3:**
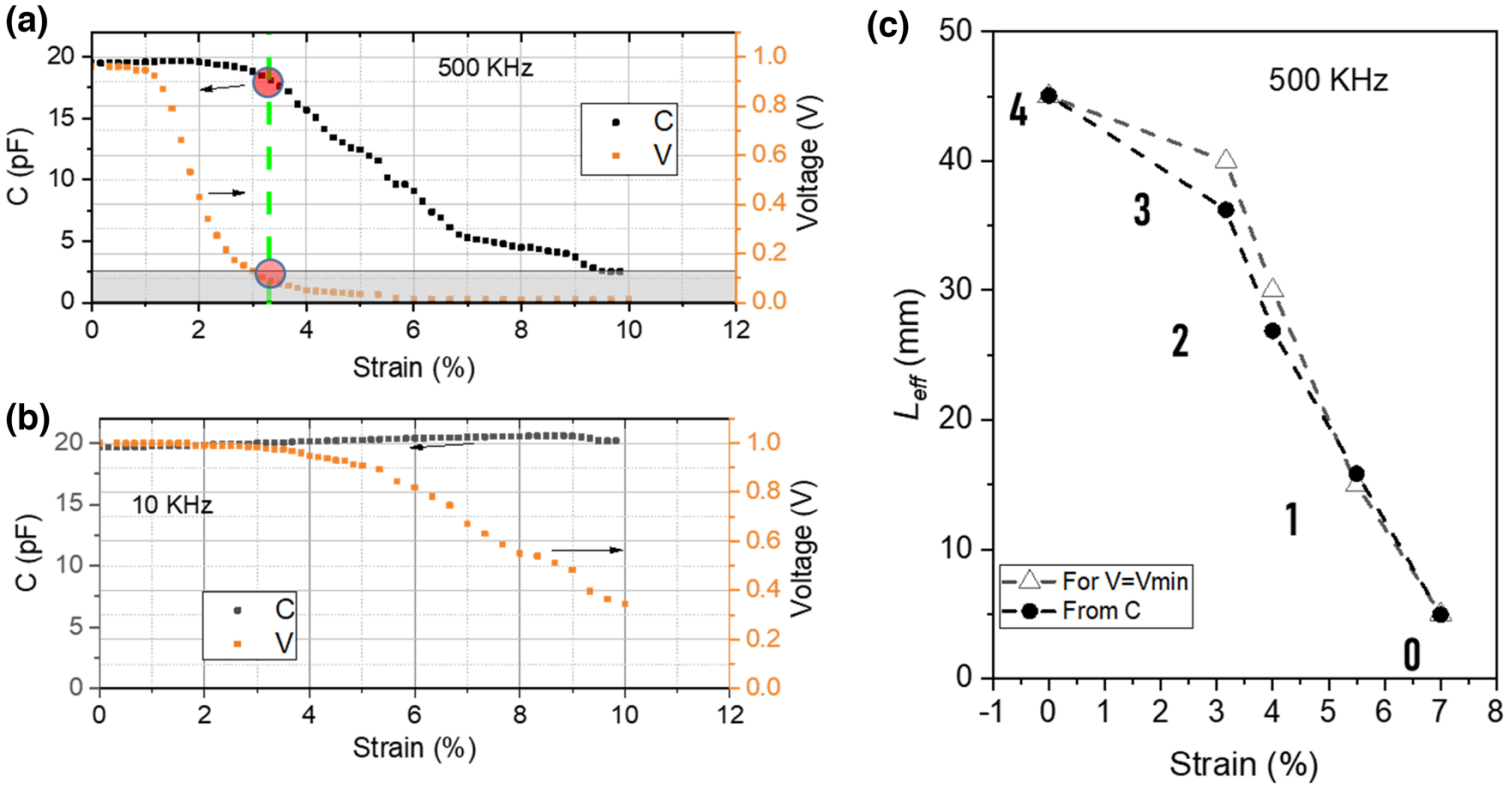
Relationship between sensor capacitance and voltage dissipation: (**a**,**b**) influence of voltage dissipation on the measured capacitance at high (500 kHz) and low (10 kHz) frequencies, respectively; the voltage is collected from the end of the sensor. (**c**) Effective length L_eff_ versus strain, derived either from the capacitance measurements or voltage disappearance.

Furthermore, we studied the relationship between the signal dissipation/strain and the sensor capacitance, as shown in Fig. 3. At a high frequency (Fig. 3a), the effective capacitance started to decrease when the voltage reached *V*_min_. This change in virtual capacitance appeared at ~ 3.2% strain, after which *L*_eff_ dropped below *L*. As expected, this behavior was absent at low interrogation frequencies. When the interrogation signal frequency was 10 kHz, the voltage attenuated but never decreased to *V*_min_; thus, the effective capacitance remained almost constant over the entire strain-loading range (Fig. 3b).

To further validate that the voltage attenuation is indeed the source of the capacitance change, we first introduce a capacitance-based effective length by calculating *L*_eff_ (*C*) from the measured capacitance *C*.

5 $$ L_{eff} (C) = \frac{{Cd_{0} }}{{e_{0} e_{r} \omega_{0} }} $$

where *e*_0_ and *e*_*r*_ are the vacuum permittivity and dielectric constant of the dielectric layer, respectively, and *ω*_0_ and *d*_0_ are the initial width and thickness (distance between both electrodes) of the dielectric layer, respectively. We also introduce a voltage-based effective length, *L*_eff_ (*V*), defined as the length between the injection point (beginning of the sensor) and the location at which the voltages reach *V*_min_. Figure 3c confirms a strong match between *L*_eff_ (V) and *L*_eff_ (C), indicating that the measured capacitance is closely related to the voltage attenuation and, therefore, to the electrode resistance. These findings makes this mechanism very interesting for distributed strain detection applications, as the effective length of the sensor can be controlled by applying an external strain.

### Accurate strain measurement

In this subsection, the above-identified mechanism, in which the effective length changes with frequency or strain amplitude, is applied to distributed strain sensing.

#### Multi-information measurement capabilities

**Figure 4:**
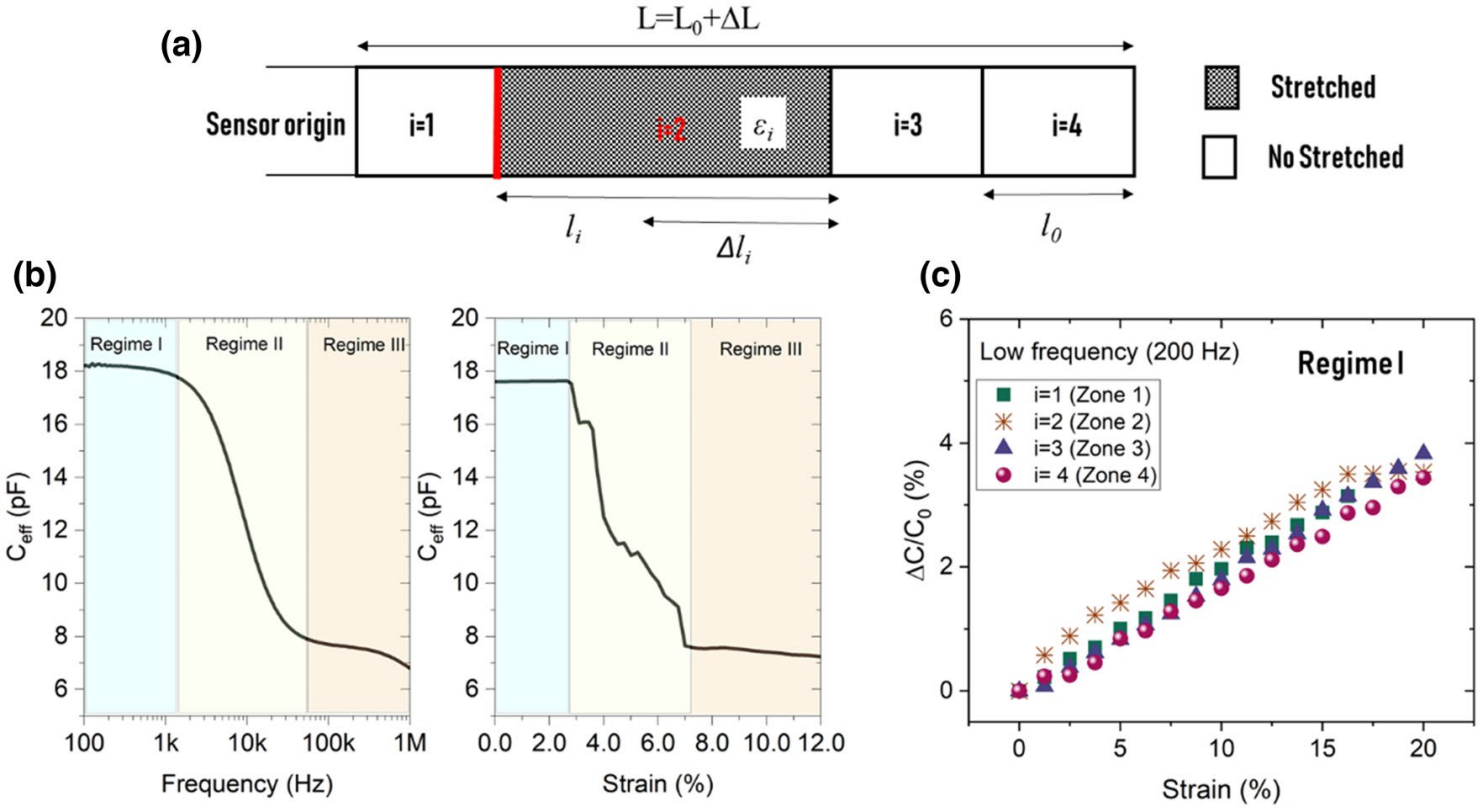
(**a**) schematic of a four-zones sensor with the main geometrical parameters. (**b**) Sensor capacitance versus frequency when zone 2 only is under strain (the behaviors when the other zones only are stretched are presented in Fig. S4) in the three regimes: the geometric regime at low *ε* and *f* (regime I), the transmission-line regime at intermediate ε and f (regime II), and the saturation regime at high ε and f (regime III). (**c**) Relative capacitance resulting from the geometric effect (length extension under stretching) vs. strain in regime I; this mechanism appears only at low frequency (200 Hz in this test).

We illustrate this concept on a sensor with four identical zones (1 ≤ *i* ≤ 4) and a length $l_{i} = \frac{L}{n}$ where *n* represents the total number of zones into which the sensor has been divided (Fig. 4a).

Based on the result of section “Signal dissipation and virtual capacitance”, we split the capacitance variation of the sensor into three independent regimes: geometric regime (regime I), transmission-line regime (regime II), and saturation regime (regime III). These three regimes appear clearly in Fig. 4b, which plots the capacitance variation when only zone #2 (*i* = 2) is under strain. Herein, the geometric regime (low *f* or *ε*) is defined from the beginning of the measurement until the beginning of the capacitance decline. The capacitance decreases from its maximum to its minimum value within the transmission-line regime (*f* = 1.5–45 kHz, *ε* = 2.8–8.0%). Once the capacitance reaches its minimum value, the saturated regime is achieved.

Each of these regimes can be used to extract different informations regarding the strain.

In regime I, the geometric effect dominates the capacitance vs. strain relationship. The strain in the stretched zone, ε_*i*_, (strain magnitude for zone #i) can be determined from the capacitance variation in this regime. Figure 4c plots the relative capacitance under gradual strain for the four zones (*i* = 1–4) at a very low frequency (200 Hz). The capacitance linearly increased with local length extension (∆*l*_*i*_) in each zone with a GF of 0.2, reflecting the geometric effect. This capacitance variation is independent of the stretching zone (strain location) as long as the zones length is identical. In reality, the stretched length might be longer than a zone (for example, zones 2 + 3 together, or zones 3 + 4 together). Determining the strain in the stretched domain requires knowing the length of this domain.

**Figure 5:**
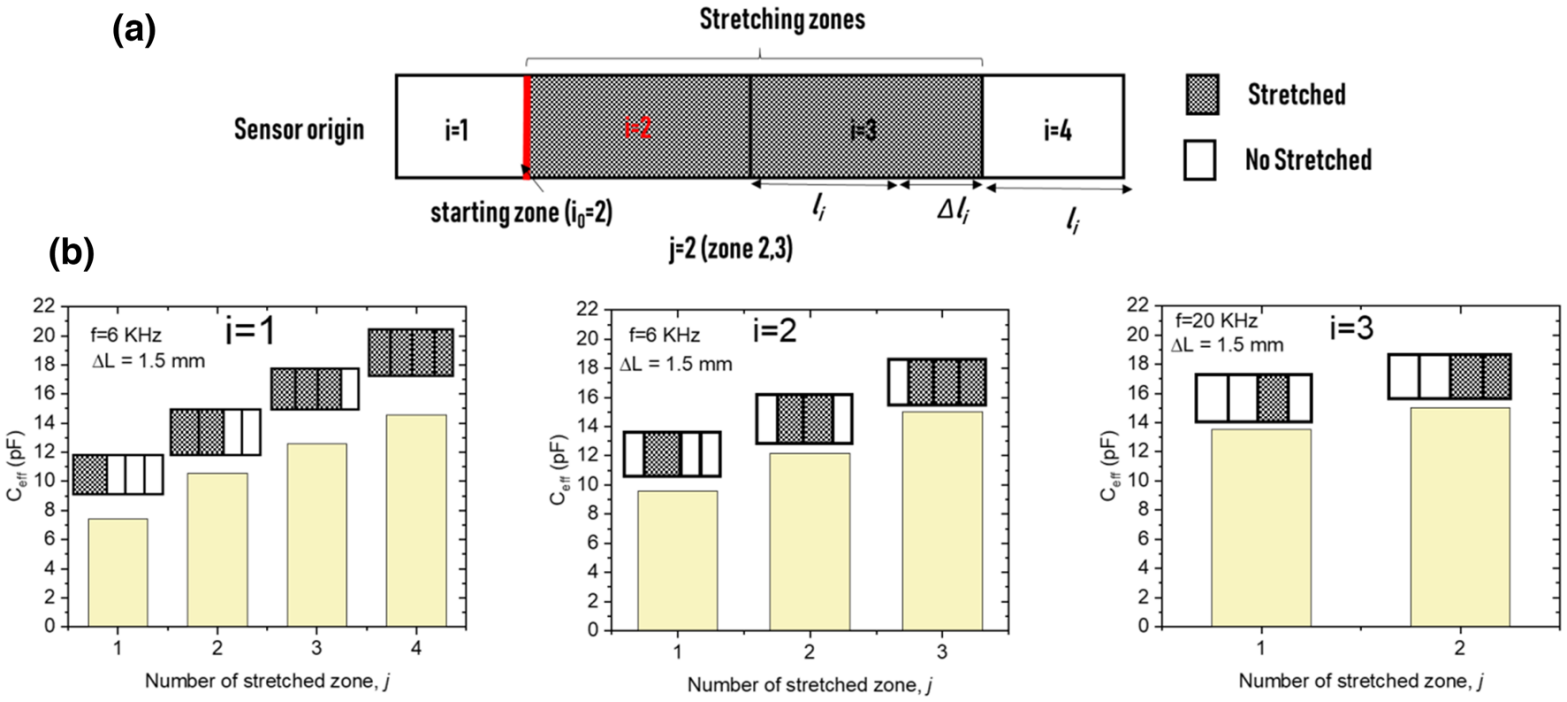
Determination of the extent of the stretched area using the transmission-line regime (regime II): (**a**) schematic of a four-zone sensor with local stretching in two zones starting in zone 2 (*i*_0_ = 2, j = 2); (**b**) charts showing the relation between the extent of the stretching area and the effective capacitance of the sensor (effective capacitance in all possible cases of *i*_0_ and j).

Regime II (the transmission-line regime) determines the length/extent of the stretched domain (*l*_*j*_), from which can be deduced the number of stretched zones (referred to as *j*). To clarify the used parameters (*j, i*_0_, *n*), we present a schematic of a four-zone sensor (*n* = 4) stretched in zones #2 and #3 (*i* = 2, 3) (Fig. 5a). In this example, *j* = 2 (hatched part) and the starting point, *i*_0_, defines the start of the stretching zone (beginning of zone 2 marked with the red line). When part of the structure is under strain, the electrode resistance is negligible in the nonstretched zones compared to that in the stretched zones. Therefore, we can assume that only the stretched length contributes to the global electrode resistance, *R*, which produces ${\text{R}}^{{\prime}} = \frac{{\text{R}}}{{\text{jL/n}}}$ in the stretched domain. Adding this new R′ into Eq. (1), the attenuation of the traveling voltage wave in the stretched domain becomes related to *j*. The voltage attenuation slope is inverse to *j*, implying that the voltage disappeared more gradually as *j* increased. This behavior is reflected in the effective capacitance, producing a relationship with *j* $(C_{eff} \sim \sqrt{\frac{j}{n}}  )$. The analytical relationship between *C*_eff_ and *j* is demonstrated in SI (Section IV). Furthermore, Fig. S3 experimentally shows the decrease of the attenuation slope of the capacitance with *j*. Figure 5b summarizes this result by showing the effective capacitance as a function of the number of stretched zones *j* for different starting points (*i*_0_) at a fixed frequency (*f* = 6 or 20 kHz) and extension (1.5 mm). Results confirmed a direct relationship between *C*_*eff*_/GF^II^ and *l*_*j*_. Note that the local strain (ε_i_) provided in the above discussion from regime I was for a sensor with unit-length zones (j = 1). We are able by integrating the result collected from regime II (values of *j*) to generalize and measure the total amount of strain applied to the sensor by replacing *j* in the following equation:

6 $$ \varepsilon = \frac{j}{n}\varepsilon_{j} $$

where ε_j_ is the local strain that depends on the stretching zone length.

The local GF_j_ also varied with *j* and remained independent to *i*_0_*,* as shown in Table 2 that lists the GF_j_s for different *j* at *i*_0_. We show that the GF_j_ is fixed at 0.78 when the entire structure was stretched (*j* = 4) and decreased progressively with j, achieving 0.2 for only one zone that was stretched (*j* = 1). This pattern of change applies to all the starting points (*i*_0_). In Section IV-2 in SI, we developed an analytical model that shows the linear relationship between GF_j_ and *j,* clarifying the sensitivity-losing source. The measurement of GF_j_ represented by the slope (angle of the fitting line) of the relative capacitance versus strain plot is shown in Fig. S3a–c (iii), where a clear dependence on *j* is present. As a result, *j* can be deduced from the degree of sensitivity of the sensor; particularly, the sensitivity linearly depended on *j*.

**Table 2:** Sensor sensitivities (GF) for all possible stretching areas in a four-zone sensor.

GF	*i*_*0*_ = 1	*i*_*0*_ = 2	*i*_*0*_ = 3	*i*_*0*_ = 4
*j* = *1*	0.2	0.18	0.2	0.2
*j* = *2*	0.33	0.38	0.38	–
*j* = *3*	0.55	0.57	–	–
*j* = *4*	0.78	–	–	–

**Figure 6:**
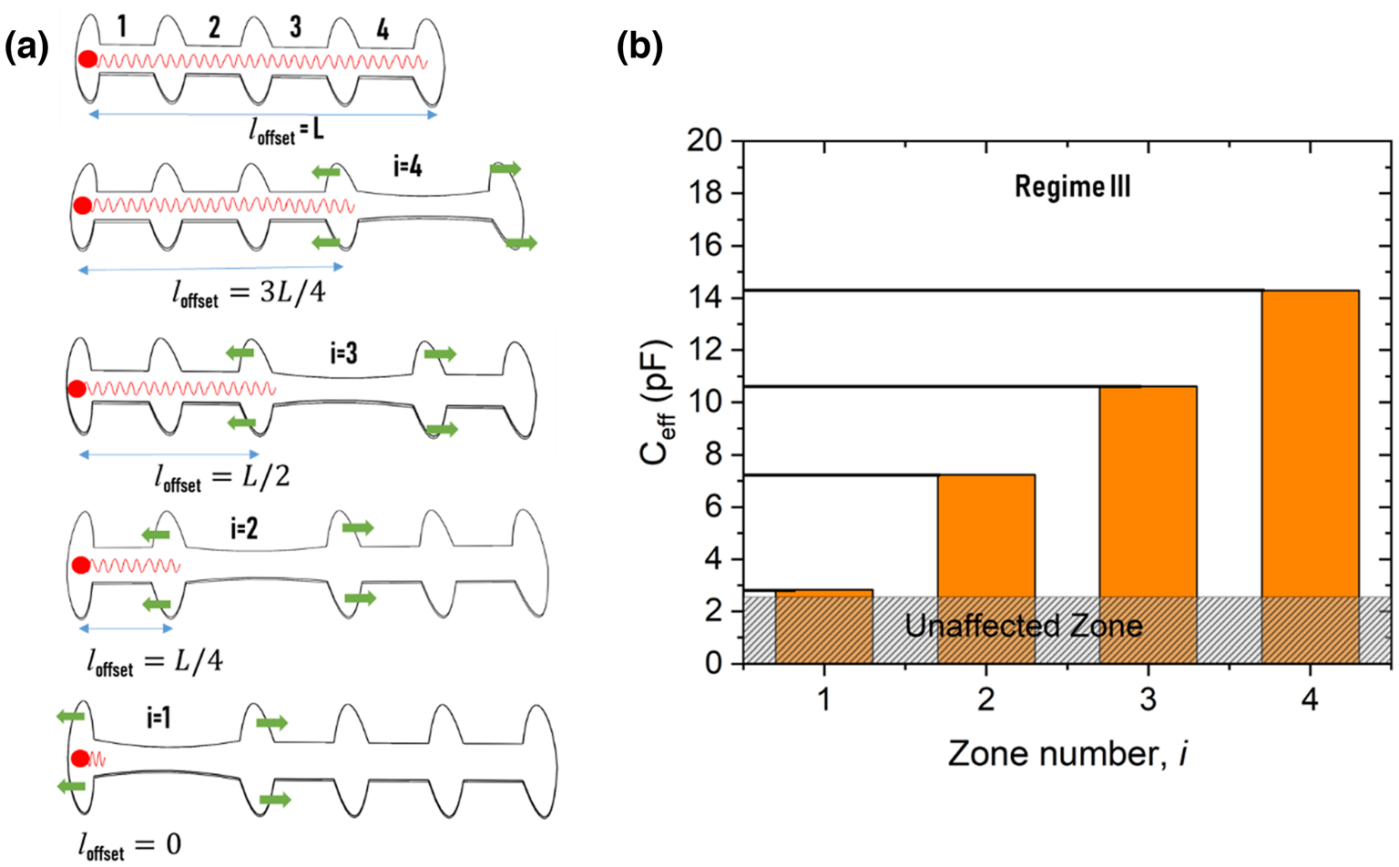
Regime III: (**a**) Schematic of signal behavior in regime III: the voltage dissipates at the beginning of the stretched zone and always dwells before the stretched zone. *l*_offset_ locates the beginning of the stretched domain; (**b**) effective capacitances at ε = 12% and f = 500 kHz when stretching different zones of the transmission line (the effective capacitance increases linearly with zone number i).

Regime III is used to identify the start of the stretching zone, *i*_*0*_, which can be defined as strain location. We measure in fact wit the regie III the size of the unaffected area or the minimum capacitance in regime III (Fig. 4b). Capacitance saturation refers to the limitation of signal penetration inside the sensor after applying a local strain (stretching one zone). The signal easily crossed the nonstretched zones as the low electrode resistance ensured no dissipation. However, as shown in Fig. 6a, when the signal reached the first high-resistance stretched zone, it dissipated and eventually faded completely. Thus, in this regime, the effective length of the sensor, represented by *l*_offset_ in Fig. 6a, corresponds to the length of all unstretched zones. The location of the strain is provided by knowing the value of *l*_offset_. For example, when zone #3 was stretched, the signal passed through the first and second zones without attenuation and was stopped at the beginning of zone #3 (Fig. 6a). Generalizing this case, we respectively deduce the zone-dependent effective length $L_{eff}^{III} (i)$ and the effective capacitance $C_{eff}^{III} (i)$ as follows:

7 $$ L_{eff}^{III} (i) = \frac{(i - 1)}{n}L = l_{offset} \quad {\text{and}} \quad C_{eff}^{III} (i) = \frac{(i - 1)}{n}C_{0} \left( {1 + \frac{{\varepsilon_{i} }}{n}} \right) $$

The above defined $L_{eff}^{III} (i)$ and $C_{eff}^{III} (i)$ concern only the effective length and effective capacitance of the sensor in regime III, respectively. Experimentally, *l*_offset_ can be deduced by measuring the effective capacitance in regime III as shown in Fig. 6b, which presents the total effective capacitance at *f* = 500 kHz when streching different zones at a strain *ε* = 12%. Herein, the capacitance reaches different values by only changing the location of the applied strain. As shown in Fig. 6b, the capacitance ranges between 2.5 pF (the capacitance of the first contact that is always present and non-zero) and 3/4 of its initial capacitance when the stretching zone is changing from #1 and #4. Here, regime #3 is reached for a high strain, but the strain threshold at which regime #3 is reached is totally programmable a priori by applying a prestretching of the sensor^32^. Doing so, opening of cracks in the electrodes will be promoted and attenuation will be achieved at a much lower strain level if required. Figure S4 shows the detailed results described above and confirms that regime III information is crucial for detecting the start of the stretched zone.

**Figure 7:**
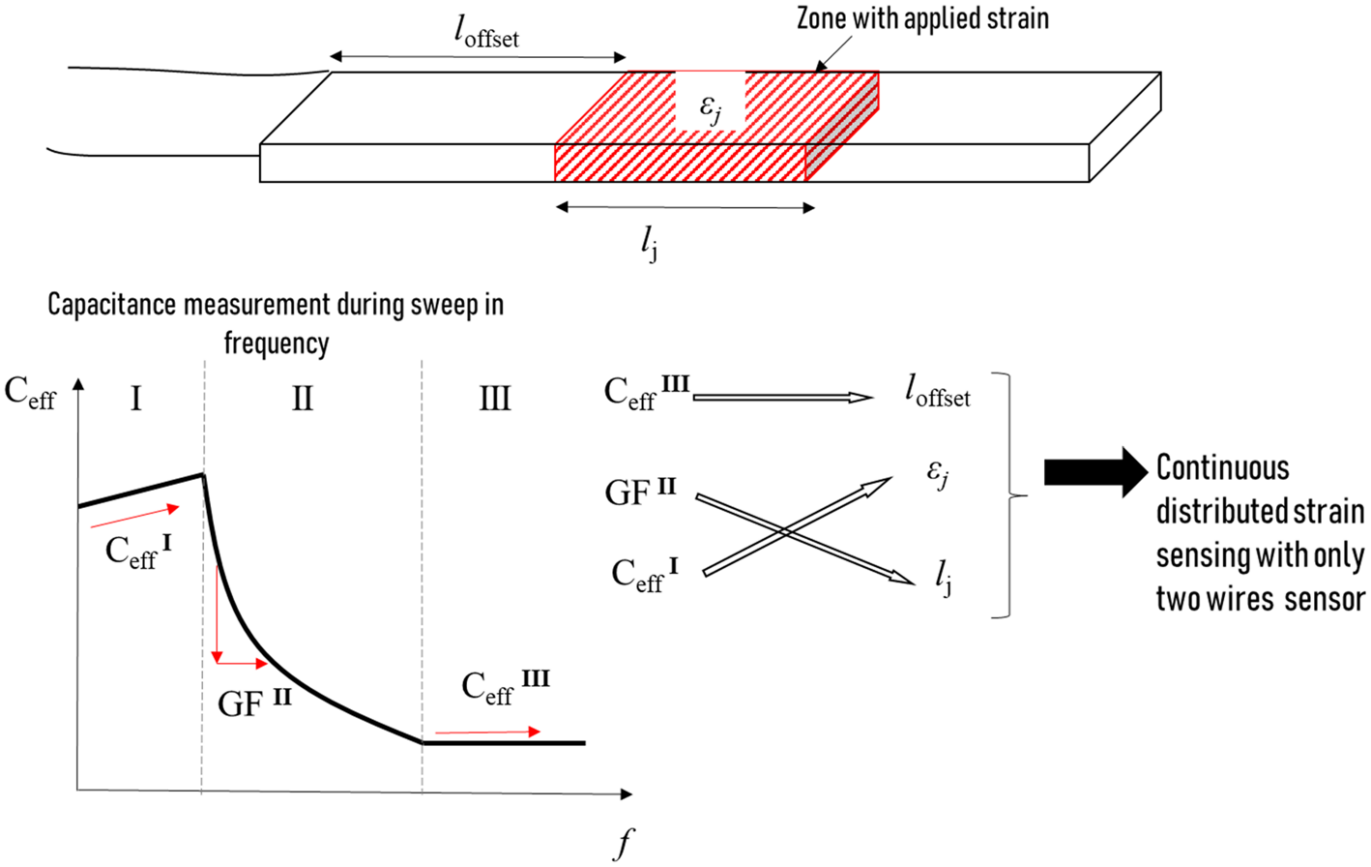
Measurement sequence and the information extractable by plotting capacitance measurements as a function of frequency. $C_{eff}^{III}$ provides information about the length between the signal injection point and the beginning of the stretching zone (*l*_*offset*_), The combination of $C_{eff}^{I}$ and GF^II^ provides information about the local strain *ε*_*j*_ and the length of the stretched domain *l*_*j*._

The results presented in this paragraph are summarized in Fig. 7. Our cracked capacitive sensor can simultaneously record the strain magnitude, strain location, and stretched area simply by measuring the sensor capacitance and selecting the right frequency. The three pieces of information simultaneously acquired by one sensor sheet enhance the sensor’s ability to obtain accurate strain information.

#### Spatial resolution improvement

In the proof of concept presented here, cracks are introduced by fragmenting the electrodes via mechanical extension. This results in a quasi-random pattern of cracks, the piezoresistivity of which is difficult to control in advance. Because of the high sensitivity of the quasi-randomly cracked electrodes, the transmission-line regime (regime II) has a very short duration. In particular, signal attenuation occurs relatively quickly in the current demonstration, making it difficult to detect the signal in many locations. To avoid this resolution limitation, the sensitivity of the electrodes might need to be reduced for some applications. By varying the number of cracks per unit length, different ranges of resistance variation (i.e. different sensitivity) can be achieved. The crack density can actually be controlled by engineering precracks (crack initiators) in the electrodes. We have already used such an approach, for example, to pattern SWCNT papers via a laser-engraving process^32^. This was investigated in the context of piezoresistive sensors; however a similar approach could be used in the present study to taylor the variation in resistance of electrodes under strain. Using this method allows the final one-sheet device to have a higher resolution than sensor arrays or independent sensor networks.

### Accurate strain-sensing applications

**Figure 8:**
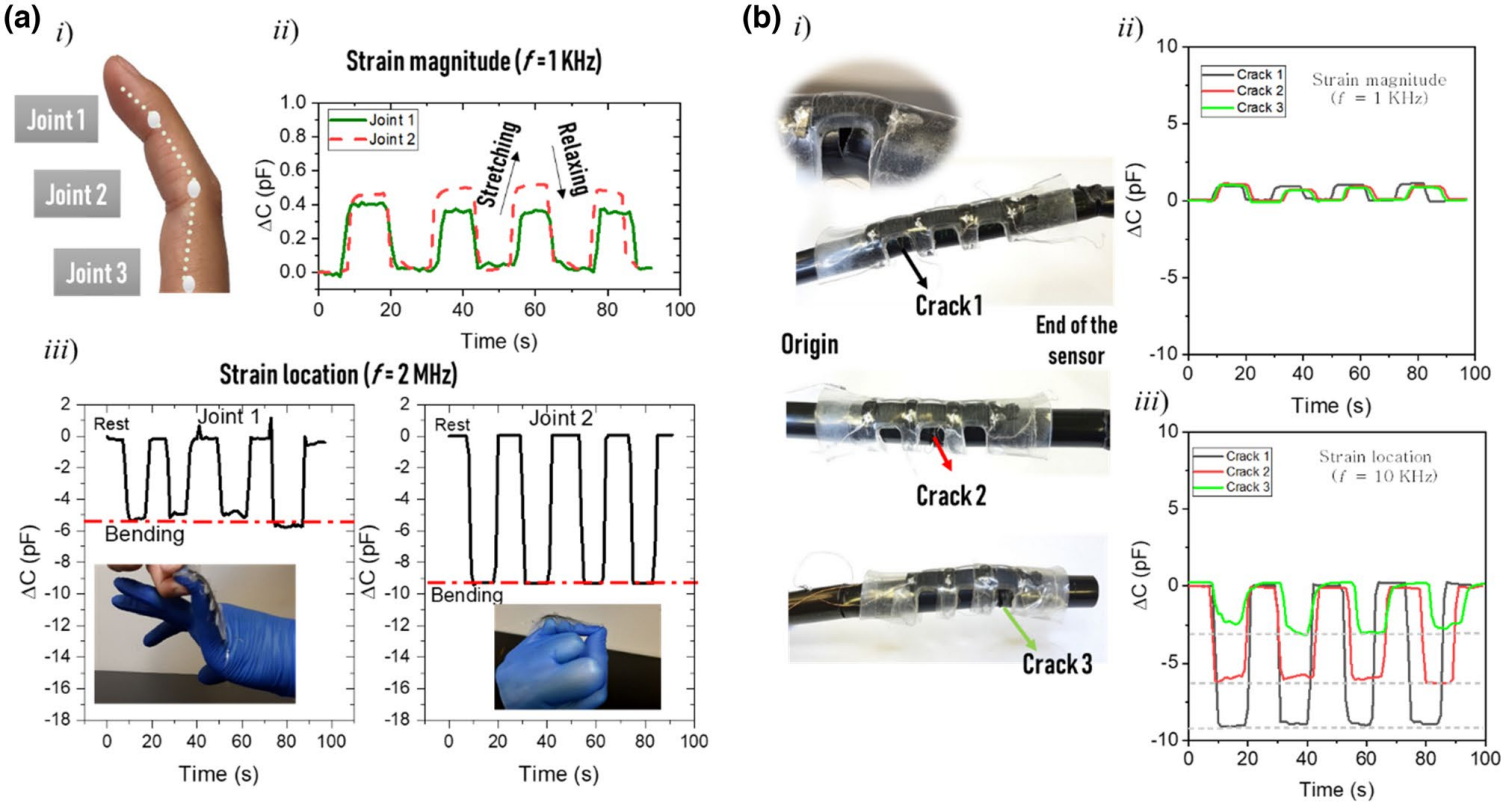
(**a**) Accurate detection of finger-joint motions, showing (i) the joints in the index finger, (ii) strain magnitudes in joints 1 and 2 during four cycles of stretching and relaxing, and (iii) identification of the bent joint from the capacity variation measured at high frequency. (**b**) Detection of crack locations and opening degrees on the nylon tube, showing (i) the photographs of the sensor implemented on a nylon tube with cracks in different locations, (ii) capacity increase realized at a low frequency (1 kHz), representing the strain magnitudes of the three cracks, and (iii) capacitance variations of the cracks at three locations, measured at a high frequency (10 kHz).

Accurate measurements of hand motions are essential for active human interactions with a virtual environment. Some of the expected future sensing applications—translating sign language into speech and text^33^, turning the hand into a gaming controller^34^, and identifying objects^35^—require a highly accurate glove that covers the entire hand. The hand is a complex structure with numerous articulated joints and degrees of freedom (see Fig. 8a.i for the joints in the index finger). For this purpose, an increasing number of smart gloves with individual or array sensors are being developed; these can be stretched to fit the finger joints and provide accurate finger-motion measurements^10,36^. However, in the existing systems, each finger requires at least three individual sensors with six cables and a complicated electronic interface, hindering hand movement. Our one-sheet sensor covers a full finger and detects accurate finger-joint motions using a minimum number of cables and rigid electronics. The strain magnitude was detectable under low-frequency operation (1 kHz) (Fig. 8a.ii), during which the capacitance increased by 0.4 pF upon bending any joint of the index finger. Meanwhile, we identify the bent joint by measuring the capacitance variation at a high frequency (2 MHz). As shown in Fig. 8a.iii, the capacitance decreased by 5.2 pF upon bending joint 1 and by 9.5 pF upon bending joint 2. To confirm the efficiency of our sensor in SHM, we also implemented our sensor on damaged nylon tubes. Nylon tubes convey compressed air, and industrial fluids as well are used in vacuum applications. They are also used in pneumatic machinery and equipment. Locating the cracks in a damaged nylon tube simplifies the equipment repair process and limits leakage issues. Figure 8b depicts a single-sheet sensor integrated on a nylon tube with multiple cracks. Crack opening increased and decreased capacitance at 1 and 10 kHz in response to local strain in a specific sensor zone. The capacitance increase (see Fig. 8b.ii) reflected the geometric variation and indicated the strain magnitude. The capacitance at 10 kHz decreased by a different level at different crack positions, allowing identification of the crack position (Fig. 8b.iii). The decrease in capacitance was more visible for cracks close to the origin than for cracks further away. For example, crack 1 caused a capacitance variation of 10 pF, whereas crack 3 (at the structure’s end) caused a capacitance variation of 2.5 pF.

## Conclusion

In this study, we presented a soft capacitive sensor that can collect accurate strain information from deformable systems. Unlike traditional sensors, our technology provides a strain sensor that can determine various strain characteristics in addition to the strain intensity. Due to the cracked electrodes, we consider the designed parallel plate capacitive sensor as a transmission line, producing an electromagnetic wave attenuation under strain. First, we clarified the relationship between electromagnetic signal dissipation (voltage) and capacitance using this model’s transmission-line properties. By injecting different interrogation frequencies in the sensor, we confirmed that our sensor is able to follow the strain continuously through a large area contrary to all existing methods, giving uniqueness to covering large areas of deformable systems, such as human motions and industrial structures, with a minimal number of individual sensors. Our sensor offers an alternative low-cost solution to mapping strain over a large area by a minimum number of wires and interrogation systems, allowing deformable systems to move freely. An impediment remains related to the rapid disappearance of the signal in the structure under a small range of strain, which limits the sensor’s spatial resolution; however, this opens the door for future work in determining the next step for controlling the amount of electrode resistance change by adjusting the crack concentration on the electrodes.

## Materials and methods

### Materials

SWCNT papers were fabricated from SWCNTs doped with 2.7% COOH groups provided by CheapTubes, Inc. The SWCNTs were more than 90-wt% pure and contained more than 5-wt% multiwalled CNTs. Their outer diameters and lengths ranged from 1 to 2 nm and from 5 to 30 µm, respectively. The CNTs were dispersed in methanesulfonic acid (CH_3_SO_3_H, Sigma Aldrich), and the stretchable dielectric material was SYLGARD 184 PDMS (Sigma Aldrich). The electrical wires were affixed to the structure using a conductive adhesive (CW2400 silver conductive epoxy from CircuitWorks^®^).

### Preparation of the SWCNT paper

The fabrication methods were reported in a previous study^29^ (refer to Fig. S5 for a pictorial summary). The CNT paper was developed via the filtration method. First, the SWCNTs (0.5 wt%) were dissolved in CH_3_SO_3_H to create a liquid solvent. The SWCNT/CH_3_SO_3_H solvent was sonicated in a Brason 8510 sonicator (250 W; Thomas Scientific) for 60 min. The mixture was restirred for 12 h at 500 rpm. A 40-g volume of the solvent dispersion was vacuum-filtered through a sintered glass filter disk of 120-mm diameter. This low-porosity filter disk (DURAN™) prevents passage of the CNTs. The SWCNTs left on the filter were washed with 200 mL of water to remove any remaining CH_3_SO_3_H. After 5 h in vacuum, we obtained free-standing SWCNT paper of 80-mm diameter and 50–100-µm thickness.

### Fabrication of the cracked strain sensor

Our capacitive strain sensor is a parallel-plate capacitor prepared by sandwiching a PDMS layer between two CNT layers and covering both CNT sides with PDMS layers. First, the SWCNT paper was cut using a laser-cutting machine (Universal Laser Systems) into a repetitive pattern of (10 × 5) mm^2^ rectangular strips separated by ellipses (10 mm major axis × 5 mm minor axis) (see Fig. 1). The PDMS was prepared by treating a mixture of curing agent and PDMS monomers (mass ratio of 1:10) in a vacuum oven (approximately − 0.94 bar) to remove air bubbles. The first strips of the laser-engraved SWCNT paper were transferred to a half-cured, 0.5-mm-thick PDMS substrate to form the bottom electrodes. A series of copper wires were placed on the ellipsoidal zones and fixed with silver epoxy. A second PDMS layer precursor of equal weight was then poured onto the two existing layers. The CNT-paper integration was repeated to produce the top electrode and its electrical connections. A third PDMS was deposited onto the previous layers to fully encapsulate the SWCNT papers. Each PDMS layer was cured at 70 °C in an oven for 2 h. The superposed layers were cut using a laser-cutting machine, finally yielding an encapsulated parallel-plate capacitor with electrical connections at different locations. Finally, the elliptically shaped connection zones were fixed by gluing a poly(methyl methacrylate) (PMMA) support at the bottom, avoiding strain in this sensitive zone. The connections at the top and bottom electrodes were aligned and evenly distributed along with the sensors.

### Experimental setup

To measure the voltage dissipation along the sensor length, one sensor end was connected to an alternating-current voltage source and the voltage was measured at the other locations. The voltage dissipation was measured at four electrical connections that were evenly distributed along the sensor at 10-mm intervals (see Fig. 2a). Note that the final structure can be a simple strip with no splitting. The input signal, *V*_*AC*_, was injected at the origin (contact 0, Fig. 1). Its magnitude (*V*_0_) was 1.0 V and frequency was high (500 kHz). To avoid small voltages near the measurement limit, we assumed that the effective length was reached when the voltage reached a minimum magnitude, *V*_min_, arbitrarily set to 0.1*V*_0_.

The capacitance was deduced from the voltage and current measurements obtained using an LCR meter (Agilent E4980A). Besides enabling capacitance measurements, the LCR meter can inject a signal with controlled frequency and amplitude into the sensor. The voltage residue along the sensor length and the electrode resistance were measured using a KEYSIGHT 34461A digital multimeter. The sample was stretched and relaxed on a 5944 Instron universal testing frame. To prevent the sample from sliding during stretching, the sample was not clamped directly but was fixed from both sides with PMMA grips (see the experimental setup in Fig. S6). To create local strain in the structure, the two grips were closely placed; their gap was equivalent to the length of the stretched zone (see Fig. S7). The length variation, ∆*L*, under stretching was experimentally measured and the strain, *ε*, was then calculated as $\varepsilon = \frac{\Delta L}{{L_{0} }}$, where *L*_0_ is the initial sensor length.

## Supplementary Information

Supplementary Information.

## Data Availability

The datasets generated and/or analyzed in this study are available from the corresponding author upon request.
