# Supplementary material for: Minimizing the wiring in distributed strain sensing using a capacitive sensor sheet with variable-resistance electrodes
Source: Sci Rep. 2022 Aug 17;12:13950. doi: 10.1038/s41598-022-18265-x (PMC9385860; doi:10.1038/s41598-022-18265-x)
Supplement: Supplementary file 1 — Supplementary Information. [file 41598_2022_18265_MOESM1_ESM.docx]

**Supporting information**

Minimizing the wiring in distributed strain sensing using a capacitive sensor sheet with variable-resistance electrodes

Hussein Nesser^1^* and Gilles Lubineau^1^*

(1) Mechanics of Composites For Energy and Mobility Lab, Mechanical Engineering Program, Physical Science and Engineering Division, King Abdullah University of Science and Technology (KAUST), Thuwal 23955-6900, Kingdom of Saudi Arabia

E-mail: hussein.nesser@kaust.edu.sa, gilles.lubineau@kaust.edu.sa

**Keywords:** distributed strain sensing, capacitive sensor, cracked electrodes, transmission line

# Electromechanical characterization

Through electromechanical measurements, we observe the interaction between the electrode resistance and sensor capacitance under a mechanical load **(Figure S1)**. The resistance *R* of the fragmented electrodes alone exponentially increased with increasing stretching extent of the sensor **(Figure S1a)**. When cracks appeared in the conductive CNT papers, the piezo-resistivity of the electrodes greatly increased. Various resistances can be achieved by increasing the number of cracks per unit length. Meanwhile, the crack density can be controlled by patterning the pre-cracks (crack initiators) under mechanical loading. Because electrodes are inherently resistive, the capacitance of the sensor behaves as a transmission line at certain strains and frequencies (**Figure S1b,c**); this capacitance behavior refers to the voltage dissipation in the structure.

*
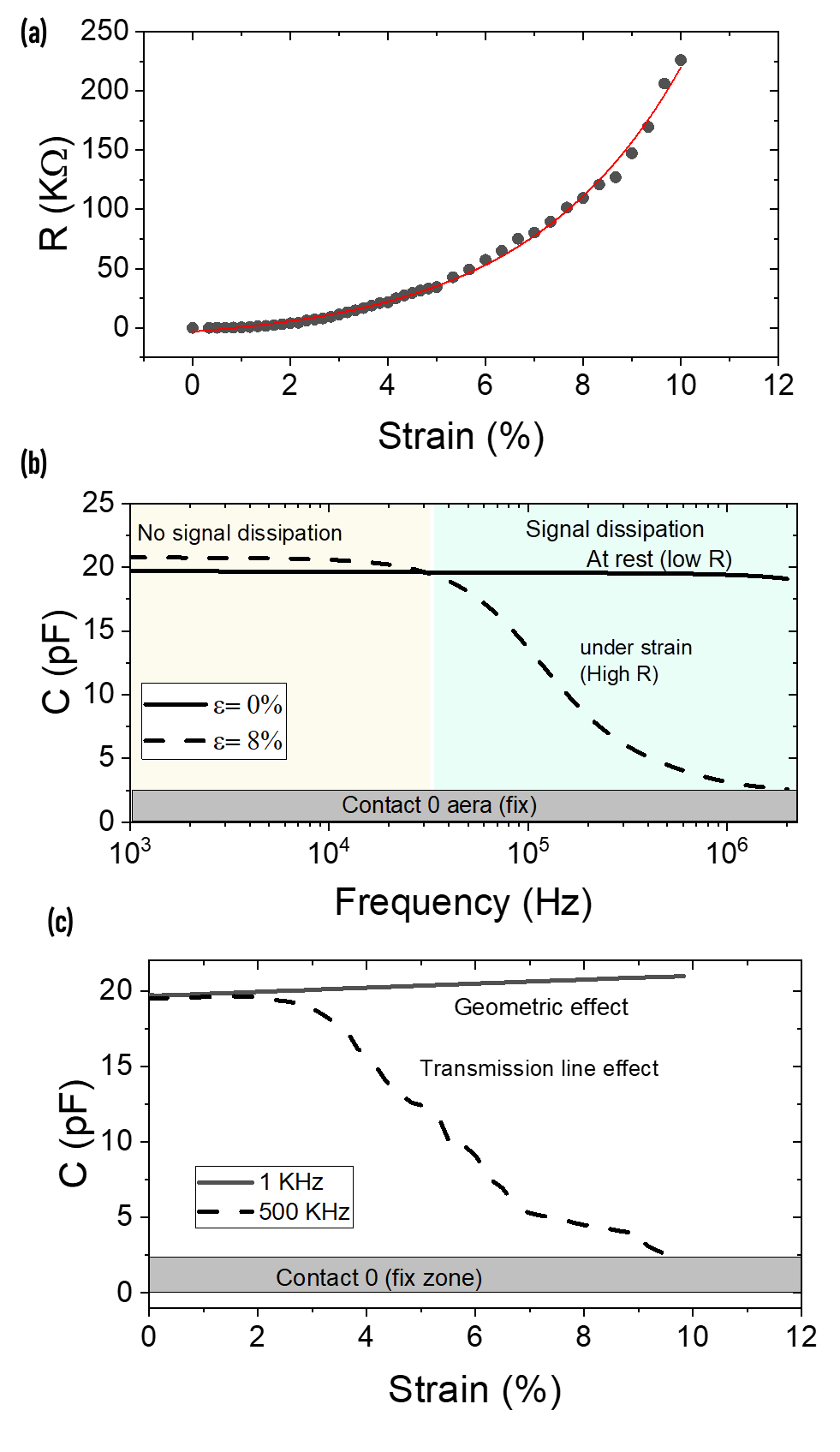
*

**Figure S1.** Electromechanical characterization of the sensor: (a) Effect of strain on the single electrode resistance; (b,c) Capacitance versus frequency and capacitance versus strain, respectively (the gray zone represents the first electrical connection area of the sensor (contact 0).

# Voltage dissipation

As shown in **Figure S1,a**, the resistance of the isolated electrode was an exponential function of strain, increasing from low resistance (<100 Ω) at low strains to 250 KΩ when the strain reached 10%. Panels b and c of **Figure S1** confirm that under low interrogation frequencies and strains, our sensor response is governed by the well-known geometrical phenomenon of the capacitance variation. At higher frequencies (>40 KHz), the capacitance significantly dropped under a strain of 8% while it remained constant at rest. **Figure S1.c** shows the capacitance-versus-strain behaviors at two frequencies. When supplied with a 1-kHz signal, the capacitance increased with a GF of 1, that is the classical change in capacitance ascribed to a classical geometrical effect. However, when supplied with a 500-kHz signal, the relative capacitance decreased by 100% after 10% strain (equivalent to a GF of 10). This capacitance behavior can be attributed to voltage dissipation in the structure.

*
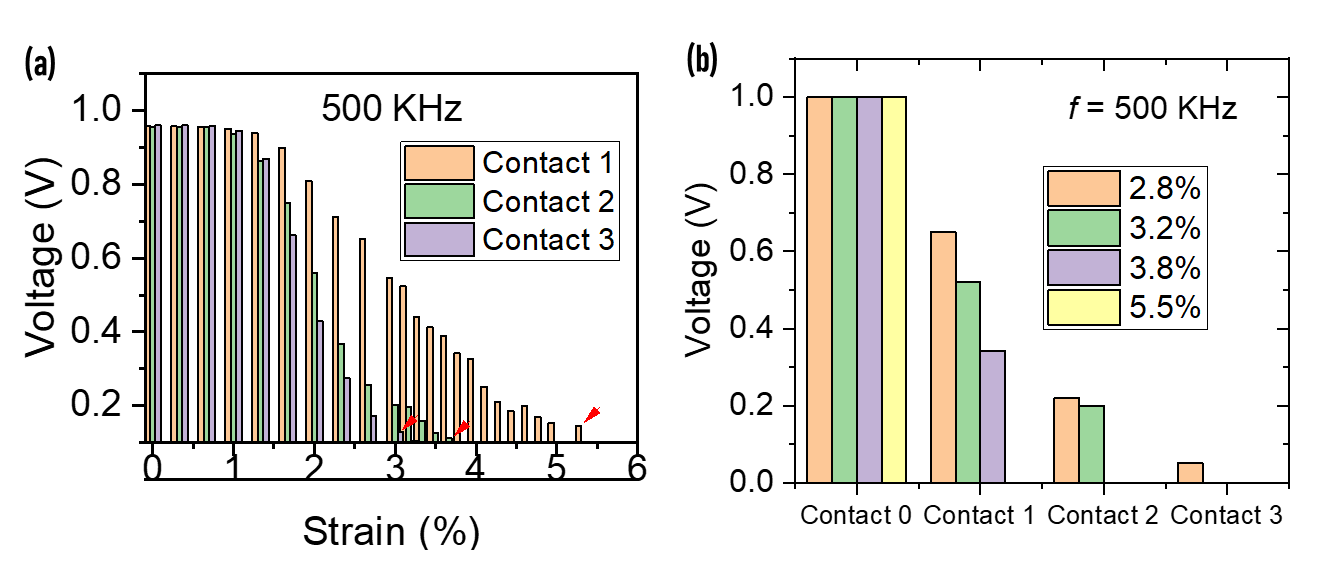
*

**Figure S2.** (a) Bar graph of voltages under different strains recorded at different locations (the red arrows point to where the voltage disappears); (b) Residual voltage at each electrical contact point under different strain levels.

As shown in **Figure S2.b**, *L*_eff_ (the effective length over which the electrical signal actually penetrates) was directly related to strain. At every z position, the signal was lost under a specific strain.

# Regime I: Strain intensity in one zone under strain

In regime I, the geometric effect dominated the capacitance-versus-strain relationship; the total capacitance is given by:

$C_{I}=e_{0}e_{r}\frac{w_{0}L}{d_{0}}$ (neglecting the effect of Poisson’s ratio). (1)

The new capacitance depends on the permittivity of the material (*e_r_*) and the geometric term ($\frac{w_{0}L}{d_{0}}$). Here, *L* is the stretched sensor length including the extended length of the stretched zone (∆l*i*). It is written as

$L=L_{0}+\Delta l_{i}$, (2)

where *L*_0_ is the initial length. The new capacitance becomes

$C_{I}=e_{0}e_{r}\frac{\omega_{0}(L_{0}+\Delta l_{i})}{d_{0}}$. (3)

Rearranging, we get

$C_{I}=e_{0}e_{r}\frac{\omega_{0}L_{0}}{d_{0}}(1+\frac{\Delta l_{i}}{L_{0}})$, (4)

$C_{I}=e_{0}e_{r}\frac{\omega_{0}L_{0}}{d_{0}}(1+\frac{\Delta l_{i}}{nl})$ (where $\varepsilon=\frac{\Delta l_{i}}{l}$). (5)

In the above expression, *n* is the total number of zones. The strain *ε* is applied only in the stretched domain *i*, so the strain is assigned only to the length of the stretched zone (unlike the capacitance, which is measured over the entire sensor length). The capacitance when the sensor is stretched in one zone for example is given by

$C_{I}=C_{0}(1+\frac{\varepsilon}{n})$, (6)

where *C*_0_ is the initial capacitance of the entire sensor. In case multiple adjacent zones are stretched simultaneously, this has to be considered in the above equation that now includes two unknowns: the stretch and the extend of the stretch area. Both can be determined by coupling this equation with observations from regimes II and III.

The sensitivity was low because the strain was applied to part of the sensor, whereas the capacitance was measured over the entire sensor (*i.e.*, the gauge factor (GF) was divided by the total zone number *n*). The above equations demonstrate the capacitance variation under a local strain in regime I depends on the total zone number *n*. More specifically, the new capacitance is inversely proportional to *n*, implying that dividing the transmission line into many zones impairs the sensor sensitivity. This weakness can be limiting for sensor applications.

# Regime II: Detecting the extent of the stretched area

#
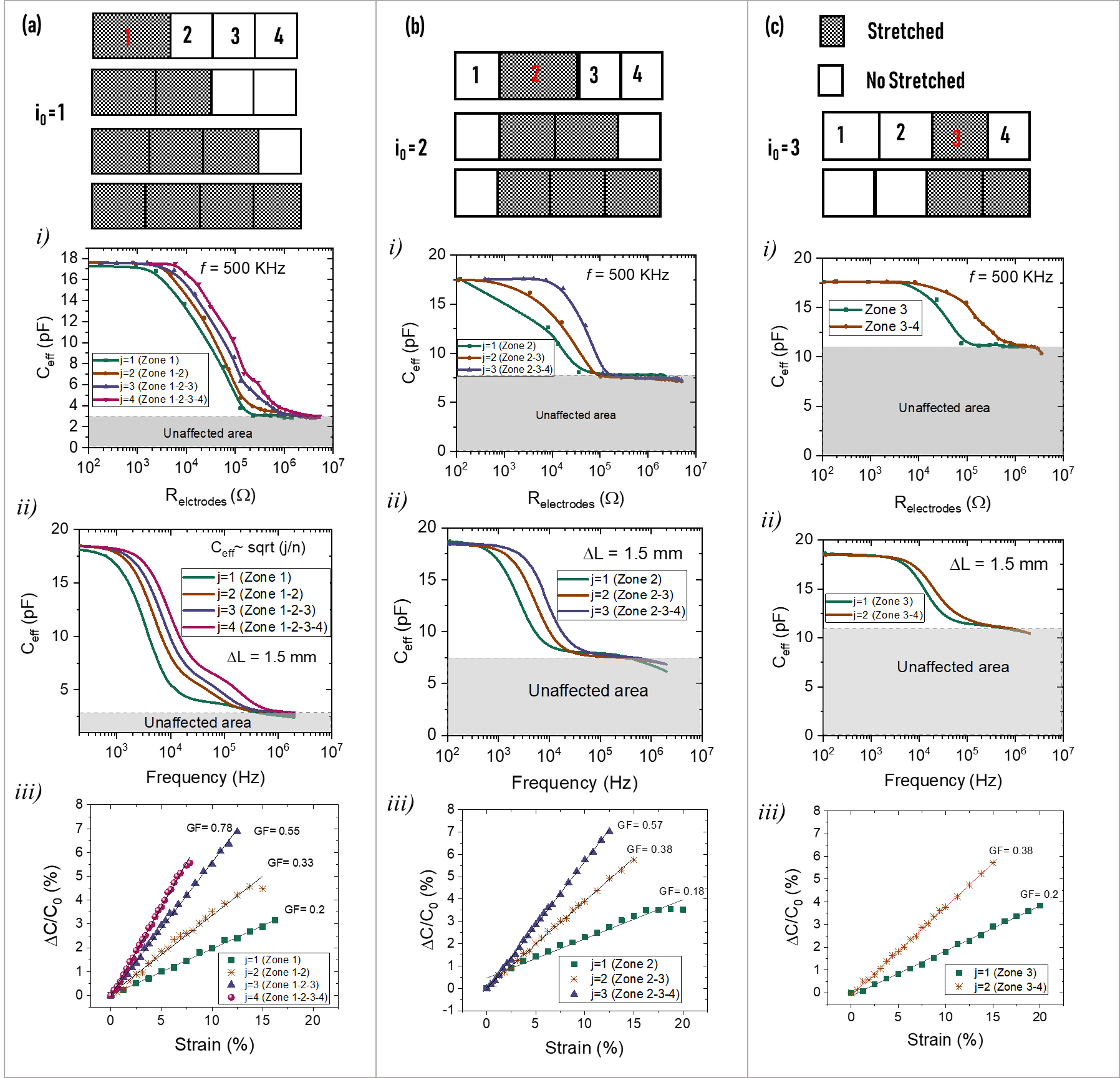


**Figure S3.** Extent of the area exposed to strain in regime II: all possible cases for (a) i_0_ = 1, (b) i_0_ = 2, and (c) i_0_ = 3. Row (i) effective capacitances as functions of electrode resistance for all j; Row (ii) effective capacitance attenuations as functions of frequency for all j; Row (iii) Relative capacitances as functions of strain for all j (the GFs are the slopes of these plots).

***1- Determining the extent of stretched area from the attenuation voltage: Relation between C and j***

In this subsection, we derive the effective capacitance *C*_eff,_*_j_* when one (or more) zones of the sensor is (are) stretched at the same time. *j* represents the number of stretched zones, *C*_eff_ denotes the effective capacitance when the whole sensor is stretched, and *C*_eff,_*_j_* denotes the effective capacitance when part of the sensor is stretched.

The voltage in a transmission line is given by

$V(z)=V_{0}e^{-\alpha z}$, (7)

where *𝑉*_0_ is the magnitude of the alternative input voltage and $\alpha=\sqrt{\pi fR^{'}C^{'}}$ is the attenuation factor, which depends on the frequency of the interrogation signal *f*, the capacitance per unit length $C'$, and the resistance of the electrode per unit length *R’*.

At *z* = *L*_eff_, *V*(*L*_eff_) = *V*_min_  with

$V_{\min}=V_{0}e^{-\alpha L_{eff}}$. (8)

*V*_min_ is the “ineffective” or “nonexistent” voltage. *L*_eff_*_,j_* is then given by

$L_{eff,j}=-\frac{\ln\left(\frac{V_{\min}}{V_{0}}\right)}{\sqrt{\pi fC^{'}R^{'}}}$. (9)

When a section of an electrode is unstretched, its resistance is negligible compared to the resistance of the stretched zones. Therefore, only the stretched length is responsible for the global electrode resistance *R*. In this stretched zone, the linear resistance and capacitance becomes: $R^{'}=\frac{R}{\frac{jL}{n}}$. and $C^{'}=\frac{C}{L}$. Here, *C* is the sensor capacitance and *R* is the electrode resistance.

In this case, the effective length of the sensor under partial stretching (when one or more zones are stretched at the same time) can be written as

$L_{eff,j}(j)=\frac{-\ln\left(\frac{V_{\min}}{V_{0}}\right)L}{\sqrt{\pi fCR}}\sqrt{\frac{j}{n}}$. (10)

Note that *L*_eff,_*_j_* = *L*_eff_ when *j* = *n* (the entire sensor is stretched)

The effective capacitance *C*_eff,_*_j_* of a partially stretched sensor is

$C_{eff,j}(j)=\frac{e_{0}e_{r}w_{0}L_{eff,j}(j)}{d_{0}}=C\frac{-\ln\left(\frac{V_{\min}}{V_{0}}\right)}{\sqrt{\pi fCR}}\sqrt{\frac{j}{n}}=C_{eff}\sqrt{\frac{j}{n}}$. (11)

According to this equation, *C*_eff,_*_j_* changes when changing the area of the stretching zone *j* and we take advantage of this to determine the extent of the stretching area.

***2- Determining the extent of the stretching area from the degree of sensitivity: relation between GF and j***

In Row (iii) of **Figure S4**, the slopes of the relative capacitance versus strain plots were given as the GFs. **Figure S4** confirms that the GF (slope) depends on the extent of the stretching area. The gauge factor ranged from 0.2 to 0.78 depending on the number of zones stretched at the same time.

The capacitive GF of a global sensor under strain is

$GF=\frac{\frac{\Delta C}{C_{0}}}{\frac{\Delta L}{L_{0}}}$, (12)

where ∆*C*/*C*_0_ is the relative capacitance variation of the global sensor and ∆*L*/*L*_0_ is the strain *ε* over the entire sensor.

The gauge factor under local strain (*GF_j_*) slightly differs from the global sensor sensitivity because the capacitance is measured over the entire sensor when the same strain is applied over different lengths (parts of the sensor). Under a constant strain, the extension *∆l_j_* of the sensor depends on *j* and is given by $\Delta l_{j}=\sum\Delta l_{i}$.

The local capacitance variation ∆*C_j_* is related to *∆l_j_* (the classical capacitance relation) and is small compared to *C*_0_. It results from stretching a small part of the “capacitor” while the rest of the capacitor is strain-free. For identical strains, we can write

$l_{0}=\frac{j}{n}L_{0}$, ${\Delta L=\Delta l}_{j}$ and $\Delta C=\Delta C_{j}$.

In this case

$\varepsilon_{j}=\frac{\Delta l_{j}}{l_{0}}=\frac{\Delta L}{\frac{j}{n}L_{0}}=\frac{n}{j}\varepsilon$ (14)

ε_j_ is the local strain refer to the zone j

The *GF_j_* can then be written as

${GF}_{j}=\frac{\frac{\Delta C}{C_{0}}}{\varepsilon_{j}}=\frac{j}{n}\frac{\frac{\Delta C}{C_{0}}}{\varepsilon}=\frac{j}{n}GF$. (15)

# Regime III: Detecting where the stretched domain starts by measuring the capacitance at high *ε* or *f*


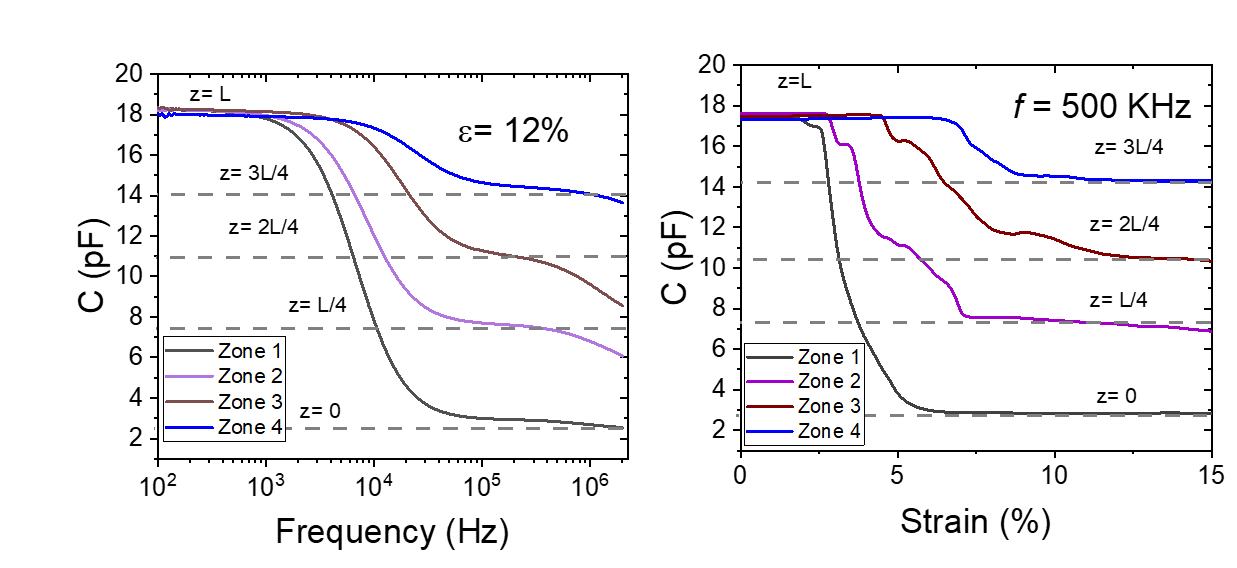


**Figure S4.** (left) Capacitances as functions of frequency under fixed strain in all zones (the constant capacitance at high frequency is different in each zone); (right) Capacitances as functions of strain at fixed frequency in all zones (the minimum capacitance under high strain is different in each stretched zone).

# Fabrication process and experimental setup


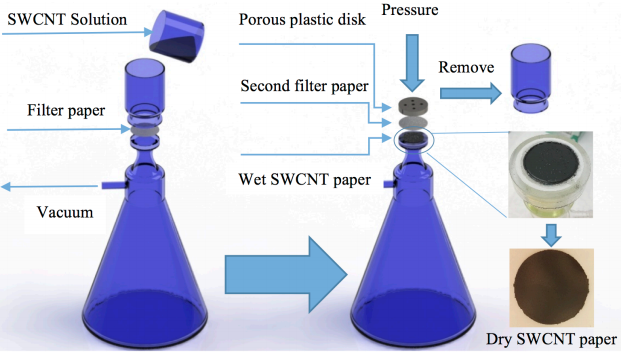


**Figure S5.** CNT-paper fabrication process based on filtration methods.


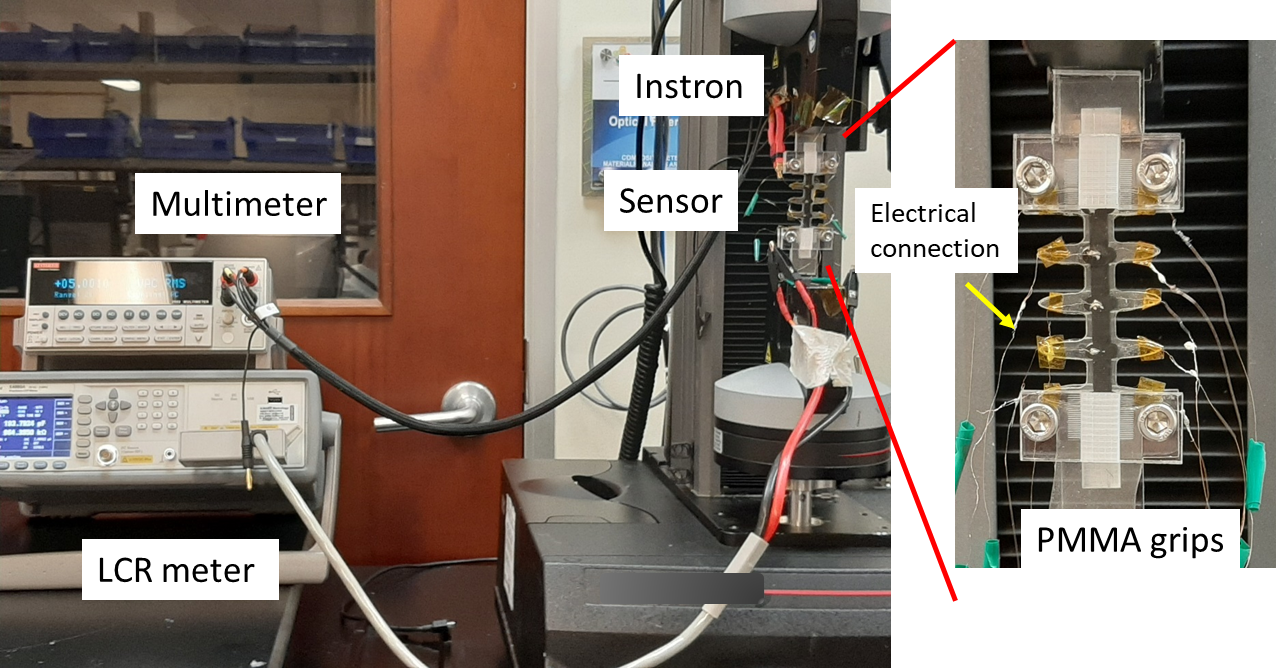


**Figure S6.** Experimental setup: The LCR meter injects a signal into the sensor. The multimeter collects the voltage residue at different locations of the stretched sensor. The sensor is stretched on an Instron universal testing frame. Right-hand-side image shows the PMMA grips clamping the sensor from both sides.


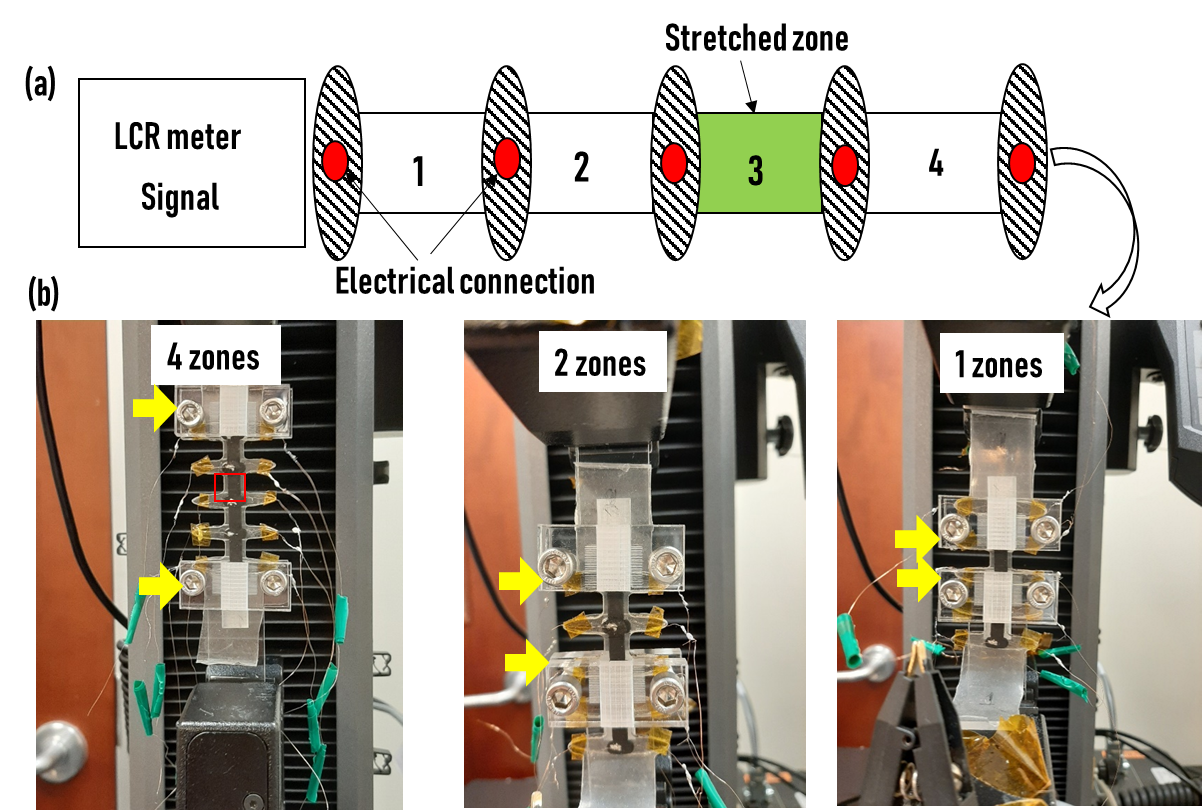


**Figure S7.** Experimental process of local strain application: (a) Schematic of a sample with one stretched zone (zone 3). The LCR meter that injects the signal and measures the effective capacitance is connected to the first electrical connection (origin). (b) Photographs showing different stretching lengths. The local strain is applied by changing the gap between the PMMA grips as only the space between the grips is subjected to strain.
